# Whole blood transcriptome analysis in dairy calves experimentally challenged with bovine herpesvirus 1 (BoHV-1) and comparison to a bovine respiratory syncytial virus (BRSV) challenge

**DOI:** 10.3389/fgene.2023.1092877

**Published:** 2023-02-17

**Authors:** Stephanie O’Donoghue, Bernadette Earley, Dayle Johnston, Matthew S. McCabe, Jae Woo Kim, Jeremy F. Taylor, Catherine Duffy, Ken Lemon, Michael McMenamy, S. Louise Cosby, Derek W. Morris, Sinéad M. Waters

**Affiliations:** ^1^ Animal and Bioscience Research Department, Animal and Grassland Research and Innovation Centre, Teagasc, Grange, Meath, Ireland; ^2^ Discipline of Biochemistry, National University of Ireland, Galway, Ireland; ^3^ Division of Animal Sciences, University of Missouri, Columbia, MO, United States; ^4^ Veterinary Sciences Division, Agri-Food and Biosciences Institute, Stormont, Belfast, Northern Ireland

**Keywords:** BoHV-1, transcriptome, whole blood, experimental challenge, BRD

## Abstract

Bovine herpesvirus 1 (BoHV-1), is associated with several clinical syndromes in cattle, among which bovine respiratory disease (BRD) is of particular significance. Despite the importance of the disease, there is a lack of information on the molecular response to infection *via* experimental challenge with BoHV-1. The objective of this study was to investigate the whole-blood transcriptome of dairy calves experimentally challenged with BoHV-1. A secondary objective was to compare the gene expression results between two separate BRD pathogens using data from a similar challenge study with BRSV. Holstein-Friesian calves (mean age (SD) = 149.2 (23.8) days; mean weight (SD) = 174.6 (21.3) kg) were either administered BoHV-1 inoculate (1 × 10^7^/mL × 8.5 mL) (*n* = 12) or were mock challenged with sterile phosphate buffered saline (*n* = 6). Clinical signs were recorded daily from day (d) −1 to d 6 (post-challenge), and whole blood was collected in Tempus RNA tubes on d six post-challenge for RNA-sequencing. There were 488 differentially expressed (DE) genes (*p* < 0.05, False Discovery rate (FDR) < 0.10, fold change ≥2) between the two treatments. Enriched KEGG pathways (*p* < 0.05, FDR <0.05); included Influenza A, Cytokine-cytokine receptor interaction and NOD-like receptor signalling. Significant gene ontology terms (*p* < 0.05, FDR <0.05) included defence response to virus and inflammatory response. Genes that are highly DE in key pathways are potential therapeutic targets for the treatment of BoHV-1 infection. A comparison to data from a similar study with BRSV identified both similarities and differences in the immune response to differing BRD pathogens.

## Introduction

Bovine respiratory disease (BRD) is a disease of multifactorial aetiology affecting cattle of all ages in Ireland (Vandermeulen et al., 2016; Murray et al., 2017; Cuevas-Gómez et al., 2020; Cuevas-Gómez et al., 2021) and internationally (Taylor et al., 2010; Baptista et al., 2017; Fernández et al., 2020), and represents a significant cause of bovine morbidity and mortality (AFBI/Department of Agriculture, 2019). Both viral and bacterial pathogens can be involved in disease onset either solely or through co-infections. Viral infections can often cause immunosuppression within the host, predisposing animals to secondary bacterial infections. Several viruses have historically been associated with BRD, including bovine viral diarrhoea virus (BVDV), bovine herpesvirus 1 (BoHV-1), bovine respiratory syncytial virus (BRSV), bovine parainfluenza virus type 3 and bovine coronavirus, while other viruses have only recently been associated with BRD (Fulton, 2020). Environmental factors such as stress induced by weaning (Johnston et al., 2016; Murray et al., 2018), transportation (Cirone et al., 2019) and thermal strain (Nonnecke et al., 2009; Louie et al., 2018) also predispose animals to disease development. Despite the use of vaccines (Chamorro and Palomares, 2020) and antimicrobials (Coetzee et al., 2019) to combat disease establishment and progression, there is a continued high prevalence of BRD globally, suggesting a gap in the knowledge surrounding the host response to infection and, in particular, the immune response to pathogen-specific infection.

RNA-sequencing (RNA-Seq) and transcriptome analysis has been used to elucidate host gene expression changes in the whole blood of animals that naturally acquired BRD (Scott et al., 2020; Sun et al., 2020; Jiminez et al., 2021; Scott et al., 2021). However, in these studies, the specific causative agent of BRD was unknown. Investigations of the host response to specific BRD pathogens using experimental challenge studies have been conducted and the protocols to induce infection following single pathogen challenge of BRD causative agents (BRSV, Infectious Bovine Rhinotracheitis (IBR), BVDV, *Mannheimia haemolytica*, *Pasteurella multocida* and *Mycoplasma bovis*) have been described in beef cattle (Gershwin et al., 2015). Tizioto et al. (2015) used RNA sequencing to examine the bronchial lymph node transcriptomes of healthy beef cattle and those infected following single challenges with BRSV, IBR, BVDV, *Pasteurella multocida*, *Mannheimia haemolytica*, or *Mycoplasma bovis* and identified differentially expressed (DE) genes involved in the bovine immune response to infection. Additionally, Behura et al. (2017) examined the role of immune tissue (healthy and lesioned lung, bronchial, retropharyngeal and nasopharyngeal lymph nodes and pharyngeal tonsil) cooperation in mounting a global immune response in beef calves following a single pathogen (BRSV, BoHV-1, BVDV, *Mannheimia haemolytica*, or *Mycoplasma bovis*) challenge. These studies described the host molecular immune response in beef cattle to infection by specific key viral and bacterial agents responsible for BRD. Johnston et al. (2019) examined gene expression changes in bronchial lymph node tissue isolated from dairy calves following a single challenge with BRSV and reported that 934 genes were DE between infected and non-infected calves and that enriched pathways were associated with the immune response. Recently, our group has also described the gene expression changes and their associated biological pathways in the whole blood of dairy calves following an infectious challenge with BRSV (Johnston et al., 2021). However, there remains a lack of information regarding the dairy animal’s host response to other important BRD causative agents, such as BoHV-1.

Second in importance to BRSV, BoHV-1 was identified as one of most commonly isolated viral agents in BRD cases in Ireland in 2019 (AFBI/Department of Agriculture, 2019). BoHV-1 is a double-stranded DNA virus of the *alphaherpesvirus* subfamily within the *varicellovirus* genus and is transmitted from animal to animal primarily through nasal and ocular secretions. BoHV-1 infection can remain latent within the ganglionic neurons (Jones, 2016; Guo et al., 2019) and animals can carry and shed the virus without exhibiting clinical symptoms, which can lead to the spread of disease.

In comparison to BRSV and other important BRD pathogens, there is a paucity of data on the host molecular response of dairy cattle to BoHV-1 infection. Therefore, our objectives were to: First, describe the clinical and haematological responses to a single pathogen BoHV-1 challenge in dairy calves; Second, examine the whole blood transcriptome response of these calves and identify the key genes and biological pathways involved in the host response; and finally, re-analyse existing whole blood RNA-Seq data from a similar study by Johnston et al. (2021) utilizing BRSV infection, using the updated ARS-UCD1.2 bovine reference genome (Rosen et al., 2020) and compare the host immune responses to BoHV-1 and BRSV.

## Materials and methods

### Preparation of BoHV-1 inoculum

Foetal calf lung (FCL) primary cells were grown in a T75 tissue culture flask in 2% Glasgow minimal essential medium (G-MEM). A 1:100 concentration solution of BoHV-1 strain 2011–415 (Esnault et al., 2022) was prepared in 2% G-MEM. The BoHV-1 dilution (5 mL) was added to the FCL cells in the T75 flask and incubated in a CO_2_ incubator at 37°C for 90 min. Following this incubation, 15 mL of G-MEM buffer was added and the solution was incubated a second time at 37°C. After 48 h, viral cytopathology of infected cells was determined *via* phase-contrast light microscopy at ×4 magnification. The flask was then stored at −80°C for 2 h, thawed and the contents transferred to sterile 50 ml centrifuge tubes. These tubes were centrifuged at 3,660 × g for 5 min, and 1 mL aliquots of supernatant were transferred to sterile 1.5 ml microcentrifuge tubes and subsequently stored at −80°C.

### Animal model

Animals were selected from a population of 43 Holstein-Friesian bull calves recruited into the study based on low BoHV-1 specific maternally derived antibody (MDA) levels and negative BoHV-1 PCR status 2 weeks prior to challenge. As the challenge and necropsy were staggered across 3 days, recruited animals (mean age 149.2 ± 23.8 days) were assigned to three groups (A, B, and C) based on sire, age and MDA levels. On the day of the challenge (day (d) 0), calves were either challenged by intranasal atomisation (Group B; *n* = 6 and Group C; *n* = 6) with a solution containing BoHV-1 (BHV-1 2011–426 strain; dose = 6.3 × 10^7^/ml × 1.35 ml per animal) or mock challenged with an intranasal atomisation of sterile phosphate buffered saline (PBS) solution (Group A; *n* = 6). Animals were restrained in a calf-restraining chute and the head was held to prevent movement during intranasal atomisation. In a previous study by our group (Esnault et al., 2022) we examined viral metagenomic sequencing on the portable, inexpensive Oxford Nanopore Technologies MinION sequencer. We assessed *in vitro* viral cell cultures and nasal swabs taken from the same calves as employed in the current study that were experimentally challenged with BoHV-1. The BoHV-1 virus was identified as the main virus in the *in vitro* cell cultures and nasal swab samples.

### Animal accommodation

The calves entered and were acclimatised to the housing environment on d −7 relative to the challenge on d 0. Two of the houses (housing groups A and B) were class 3 animal houses which were identical in layout (10.03 m × 5.01 m) while the third house was an older, separate house (6.65 m × 3.70 m). The floors were covered with sawdust and a calf-restraining chute was contained within each of the calf houses.

### Animal diet

Prior to the trial, from arrival to Agri-Food and Biosciences Institute (AFBI) Stormont, Belfast, Northern Ireland at 3 weeks of age, the calves were reared indoors and received 2 feeds per day of 2 L of 23% protein, 23% fat, calf milk replacer (Thompsons high fat; Trouw Nutrition Limited), *ad libitum* silage and approximately 200 g of calf weaner nuts (Calf Pride Weaner Mix; John Thompson and Sons, Limited), to encourage them to start eating concentrates. They were weaned from the calf milk replacer at 8–10 weeks of age and subsequently fed *ad libitum* silage and approximately 1.5 kg per day of Calf Pride Rearing Nuts, which was increased to 2 kg per day as the calves grew. For the duration of the trial, the calves had *ad libitum* access to water and silage and were fed 2 kg concentrates (17% crude protein, 4% crude oil, 9.5% crude fibre, 7.5% crude ash, 0.28% magnesium, 0.28% sodium) (Calf Pride Rearing Nuts; John Thompson and Sons, Limited) per day.

### Animal sampling

A 9 ml K_3_ EDTA blood tube of whole blood was collected daily *via* the jugular vein, with tail bleeding performed if required (i.e., if unable to collect from jugular vein) from d −1 to the day of slaughter (d 6 post-challenge), gently inverted several times and placed on ice. Whole blood was analysed for haematological variables (white blood cell (WBC) count, neutrophil percentage, lymphocyte percentage, monocyte percentage and eosinophil percentage) on an ABAXIS H5 haematology analyser (ABRAXIS Model: H5 S/N: 364372) immediately following collection.

Daily, from d −1 to d 6 (post-challenge), clinical signs (nasal discharge, ocular discharge, general demeanour, size of mandibular lymph nodes, presence of a cough, respiratory rate, respiratory character, mouth breathing, dyspnoea, presence of an expiratory grunt and rectal temperature) were recorded and scored by a veterinarian, who was blinded to the calves’ treatment status (BoHV-1 challenged or control), using a clinical scoring system similar to that previously used (Johnston et al., 2019), and previously described by Gershwin et al. (2015). Using this scoring system, points were allotted for each abnormal clinical symptom and the total number of points corresponded to the severity of disease, such that higher clinical scores were associated with more severe BRD.

### Blood sample collection for downstream RNA-Seq analysis

On day 6 of the challenge, immediately prior to euthanasia, whole blood was collected in Tempus tubes, which were snap frozen, placed on dry ice and eventually stored at −80°C until analysis. Animals were euthanised by captive bolt across 3 days with group A (control group), group B (challenge group) and group C (challenge group) animals euthanised the first, second, and third day, respectively. Lungs were scored for lesions by a qualified veterinary pathologist using the AFBI scoring system (Johnston et al., 2019), which evaluates the percentage of lesions of the total lung area and on the component parts of the lung. From the lung scoring system used, the lesions were assessed and described as; acute bronchopneumonia, subacute fibrinopurulent bronchopneumonia, percentage of pneumonic tissue, interstitial oedema, abscesses, necrotic foci, haemorrhage, and others such as pleuritis and emphysema. Day 6 post-challenge was the time at which BoHV-1 infection was considered to be at its peak as previously demonstrated by Gershwin et al. (2015). For this reason, blood samples collected on d 6 were chosen for downstream RNA-Seq analysis.

### Clinical score, lung score and haematology data analysis

Clinical scores, rectal temperature and haematology variables were analysed using repeated measures mixed models (MIXED procedure of SAS v 9.4) where time-point defined the repeated measure. Data were first assessed for normality using PROC REG and PROC UNIVARIATE. All data except the monocyte percentage were found to be normally distributed. Monocyte percentage data were Box-Cox transformed using the TRANSREG procedure of SAS ( $\hat{\lambda }$ = 2). Treatment (BoHV-1 challenged or control), time-point (day relative to challenge) and their interactions were included as fixed effects. Calf was included as a random effect. A Tukey adjustment was used to correct for multiple testing.

Lung scores (overall lung score and the percentage of the right cranial lobe lesioned) were assessed for normality using PROC REG and PROC UNIVARIATE and analysed using a mixed model ANOVA (MIXED procedure of SAS v 9.4) with treatment (BoHV-1 challenged or control) included as a fixed effect. The presence or absence of lung lesions was analysed using a Fisher’s Exact test in SAS 9.4.

### RNA extraction

Total RNA was extracted using the Tempus Spin RNA Isolation kit (Biosciences, Dublin, Ireland) according to the manufacturer’s instructions. RNA concentration was measured using the Nanodrop spectrophotometer, which determines concentration by measuring absorbance at 260 nm. RNA quality was determined using the Agilent 2,100 Bioanalyser (Agilent Technologies Ireland Ltd.,; Dublin, Ireland) with the RNA 6000 Nano LabChip kit (Agilent Technologies Ireland Ltd.,; Dublin, Ireland). The RNA integrity number (RIN) was obtained and samples had a mean RIN ± s.d. = 9.3 ± 0.26.

### RNA library preparation and sequencing

Library preparation and RNA-Seq were performed at the University of Missouri’s Genomics Technology Core and RNA samples were shipped frozen at −80°C on dry ice. RNA-Seq library preparation was performed using the TruSeq stranded mRNA Kit (Illumina, San Diego, California, United States) and high-throughput sequencing undertaken (100 bp paired-end) on an Illumina NovaSeq 6000. All sequence data produced in this study have been deposited to NCBI GEO repository and are available through the series accession number GSE199108 https://www.ncbi.nlm.nih.gov/geo/query/acc.cgi?acc=GSE199108.

### Bioinformatics and differential expression analysis

An average of 74, 303, 802 sequence reads per sample were generated in FASTQ format. Following quality and adapter trimming, 68, 915, 555 reads remained and 87.06% were uniquely mapped to the ARS-UCD1.2 bovine reference genome. Quality assessment was performed using FastQC (version0.11.8) https://www.bioinformatics.babraham.ac.uk/projects/fastqc. Reads were trimmed at the 3' end for Illumina adapters, and low quality reads (quality score <20), short reads (reads <10 bases in length), ambiguous nucleotides and poly-G-artefacts as a result of the two-colour chemistry, as used by the NovaSeq 6000 platform, were filtered using CutAdapt (version 1.18) (Martin, 2011). The quality of the trimmed reads was re-assessed using FastQC (version 0.11.8). All reads passed the basic quality statistics, except for one of the blood samples from the control group, due to an insufficient yield of total RNA. This sample was removed from subsequent analysis.

Sequence reads were aligned to the ARS-UCD1.2 bovine reference genome (Rosen et al., 2020) and read counts were generated by the conversion of aligned reads into counts per gene using the STAR (Spliced Transcripts Alignment to a Reference) alignment tool (version 2.6.1b). Differential expression analysis was carried out using the R (version 3.6.3 (2020) (R-Core-Team, 2016) Bioconductor package EdgeR (version 3.28.1) which uses an over-dispersed model to account for biological and technical variation (Robinson et al., 2010). Lowly expressed genes, considered as any gene with less than one count per million reads in at least five of the samples, were removed from the analysis. Data were normalised using the trimmed mean of M-values normalisation method and dispersions estimated using the quantile-adjusted conditional maximum likelihood (qCML) common and tagwise dispersions. Exact tests were used to detect differentially expressed genes (DEGs) between the two treatment groups, with genes categorised as DEGs if they had a Benjamini-Hochberg false discovery rate (FDR) of ≤0.1 and a fold change of ≥2.

### Pathway and gene ontology analysis

DEGs between the BoHV-1 challenged and control calves were input into the Bioconductor package ClusterProfiler (version 3.14.3) in R for Database for Annotation, Visualization and Integrated Discovery (DAVID) pathway and Gene Ontology (GO) analysis. DAVID is a significant source for any evaluation of high-throughput gene expression profiles (Huang et al., 2009a; Huang et al., 2009b). The annotation types interrogated included GOTERM_BP_ALL, GOTERM_CC_ALL GOTERM_MF_ALL and KEGG_PATHWAY. Resulting pathways and GO terms with a *p*-value <0.05 and an FDR <0.05 were considered enriched. To further examine enriched biological processes, DEGs were input into the Qiagen Ingenuity Pathway Analysis (IPA) platform and analysed according to manufacturer’s instructions (Krämer et al., 2014). IPA, through use of a repository containing biological and chemical findings termed the Ingenuity Knowledge Base, searches for targeted information on genes and proteins, as well as diseases, drugs, and chemicals (Krämer et al., 2014).

### Re-analysis of the BRSV whole blood RNA-Seq data from Johnson et al*.* (2021)

Following RNA-Seq of the 18 animals (control *n* = 6; challenged *n* = 12), an average of 41, 242, 289 sequence reads per sample were generated in FASTQ format. A total average of 40, 678, 927 reads remained following quality and adapter trimming and 83.5% were uniquely mapped to the ARS-UCD1.2 bovine reference genome. Sequence reads from the BRSV challenge were re-analysed using the same procedures, from quality control to pathway and gene ontology analysis, as described above. These data were first published by Johnston et al. (2021) using an alignment to the UMD3.1 reference genome and a new assembly and annotation of the bovine reference genome have since become available. Consequently, we re-analysed these data using the ARS-UCD1.2 reference genome to enable a better comparison of the whole blood transcriptomes of Holstein-Friesian dairy calves challenged with BRSV or BoHV-1, acknowledging that slightly different sequencing technologies were used in each study. FASTQ files are available at the NCBI GEO repository under accession number GSE152959. A comparative analysis between the BRSV and BoHV-1 challenged animals was performed using IPA and canonical pathways, upstream analysis and diseases and functions were explored. Using Z scores, the inhibition/activation states of the various pathways and molecules were determined, revealing similarities and differences between the experimental conditions. Pathway and GO analysis was performed using DAVID for DEGs that were in common (FDR ≤0.1 and fold change ≥2) and that had the same direction of effect (upregulated/downregulated) in the BoHV-1 and the BRSV data. An analysis was conducted using the Graeber labs hypergeometric calculator (https://systems.crump.ucla.edu/hypergeometric/) to determine if the similarities in gene expression patterns between the two challenge models were greater than would be expected to occur by chance alone. Additionally, DEGs common to both pathogens and unique to either BoHV-1 or BRSV were inputted into DAVID for pathway and ontology analysis (*p* ≤ 0.05, FDR ≤0.05).

## Results

### Clinical scores

**FIGURE 1:**
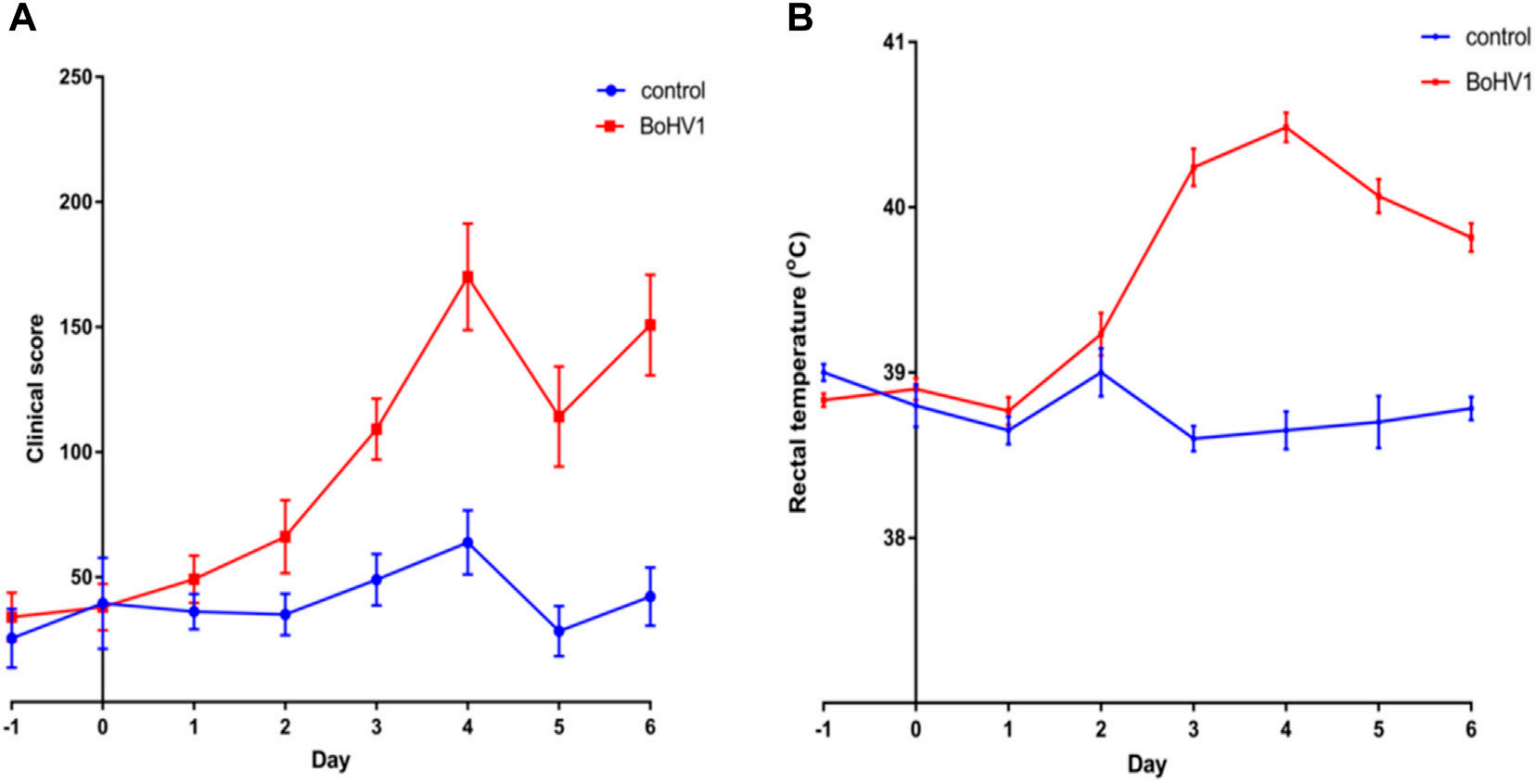
**(A)** Clinical scores from the BoHV-1 challenged (*n* = 12) and the control (*n* = 6) calves during the BoHV-1 challenge study (means and their standard errors are presented). The day of the challenge is represented as day 0. **(B)** Rectal temperatures from the BoHV-1 challenged (*n* = 12) and control calves (*n* = 6) calves over the course of the BoHV-1 challenge study (means and their standard errors are presented). The day of the challenge is represented as day 0.

Both clinical scores and rectal temperatures differed between BoHV-1 challenged and control calves with a significant treatment (BoHV-1 challenged *versus* control) × day interaction (*p* < .0001) (Figure 1A; Figure 1B). Clinical scores were greater for BoHV-1 challenged calves on d 3, 4, 5, and 6, compared to d −1 (*p* < 0.001) and rectal temperature was higher in BoHV-1 challenged calves on d 3, 4, 5, and 6 relative to d −1 (*p* < 0.0001). Clinical scores were greater in BoHV-1 challenged *versus* control calves on d 4, 5, and 6 post-challenge (*p* < 0.05). Furthermore, there was an interaction between day and treatment (*p* < 0.0001) with BoHV-1 challenged calves having higher rectal temperatures on d 3, 4, 5, and 6, compared to control calves (*p* < 0.01).

### Haematology variables

There was a treatment × day interaction for WBC count (*p* < 0.01) with a greater WBC count in controls compared with BoHV-1 challenged calves on d 2 (Supplementary Figure S1A). There was an effect of day (*p* < 0.001) on lymphocyte percentage with a lower lymphocyte percentage on d 4 relative to d −1 for all calves (*p* < 0.05) (Supplementary Figure S1B) and higher lymphocyte percentage on day 1 relative to d 3 and d 4 for all calves (*p* < 0.05). Neutrophil percentage (*p* < 0.0001) for all calves was greater on d 3 and 4 relative to d −1 (Supplementary Figure S1C). There was a treatment (*p* = 0.01) and a day effect (*p* = 0.05) and no treatment × day interaction (*p* = 0.12) for monocyte percentage (Supplementary Figure S1D). There was an effect of day on eosinophil percentage (*p* = 0.003) and no treatment × day interaction (*p* = 0.36) (Supplementary Figure S1E).

### Lung pathology

There were no differences in overall lung scores or the right cranial lobe lung scores between BoHV-1 challenged and control calves (*p* > 0.05). Despite a lack of statistical significance in the lung scores between the control and challenged calves, pathological observations showed an increased level of consolidation and lesion formation in the lungs of calves challenged with BoHV-1. In addition, an increase in the size of lymph nodes (mediastinal, retropharyngeal, and mesenteric) was evident across the majority of challenged animals, a phenomenon not as widely observed in the control animals.

### Differential gene expression and functional annotation

**FIGURE 2:**
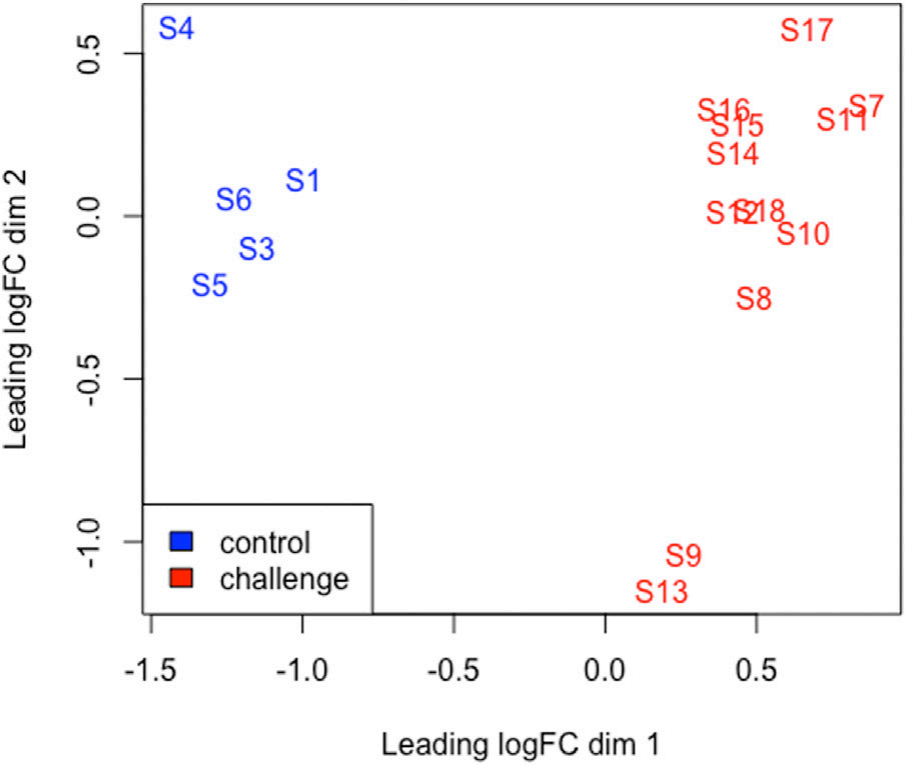
An MDS plot generated using EdgeR in R displaying the similarity of the samples. Samples from the BoHV-1 challenged calves are coloured red and control samples are coloured blue. The numbers (1-18) refer to the calf ID. Sample (S); S1-S6 (control) S7-S18 (challenge).

**FIGURE 3:**
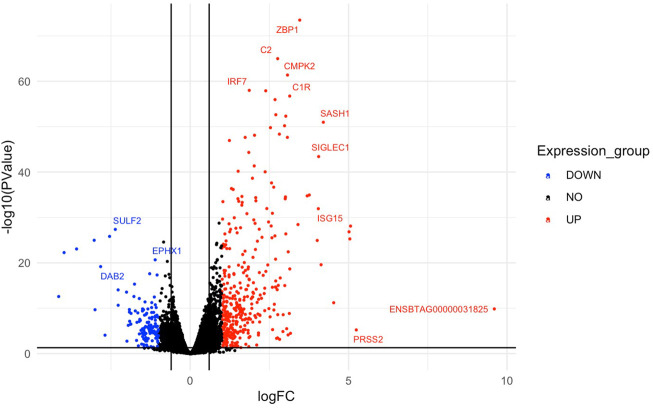
A volcano plot representing the top upregulated and downregulated genes based on log fold change values. Red dots represent the downregulated genes and upregulated genes are represented by blue dots. Genes that did not meet the threshold for differential expression are shown by the black dots in the centre of the plot. Gene symbols are assigned to the top DEGs.

**FIGURE 4:**
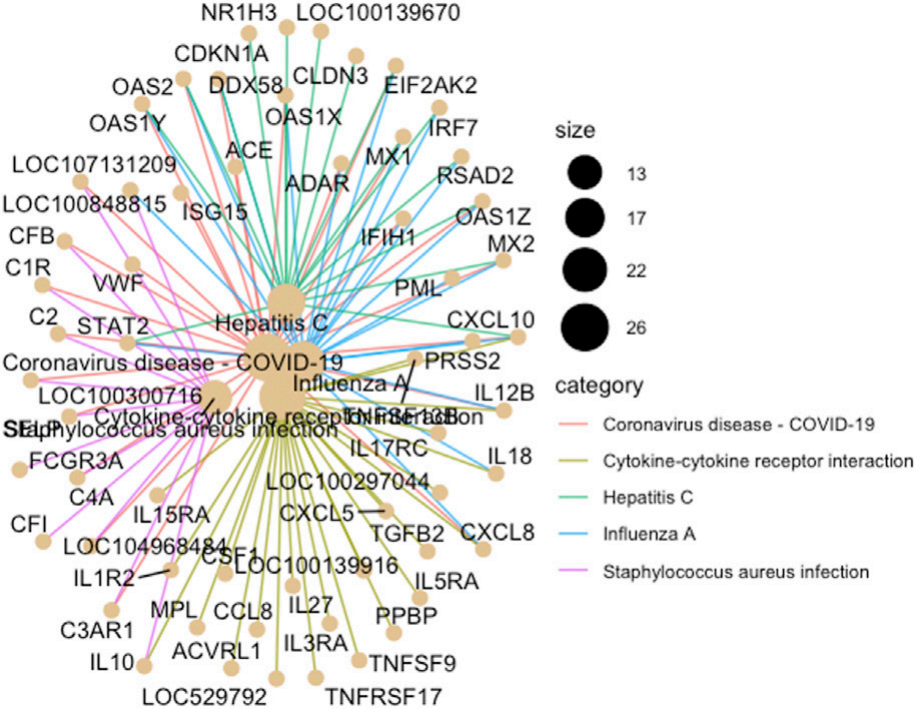
A cnet plot, made using ClusterProfiler in R, illustrating enriched KEGG pathways and gene networks. The size of the bubble at each pathway name corresponds to the number of associated genes (i.e., the larger the bubble the greater the number of genes).

**FIGURE 5:**
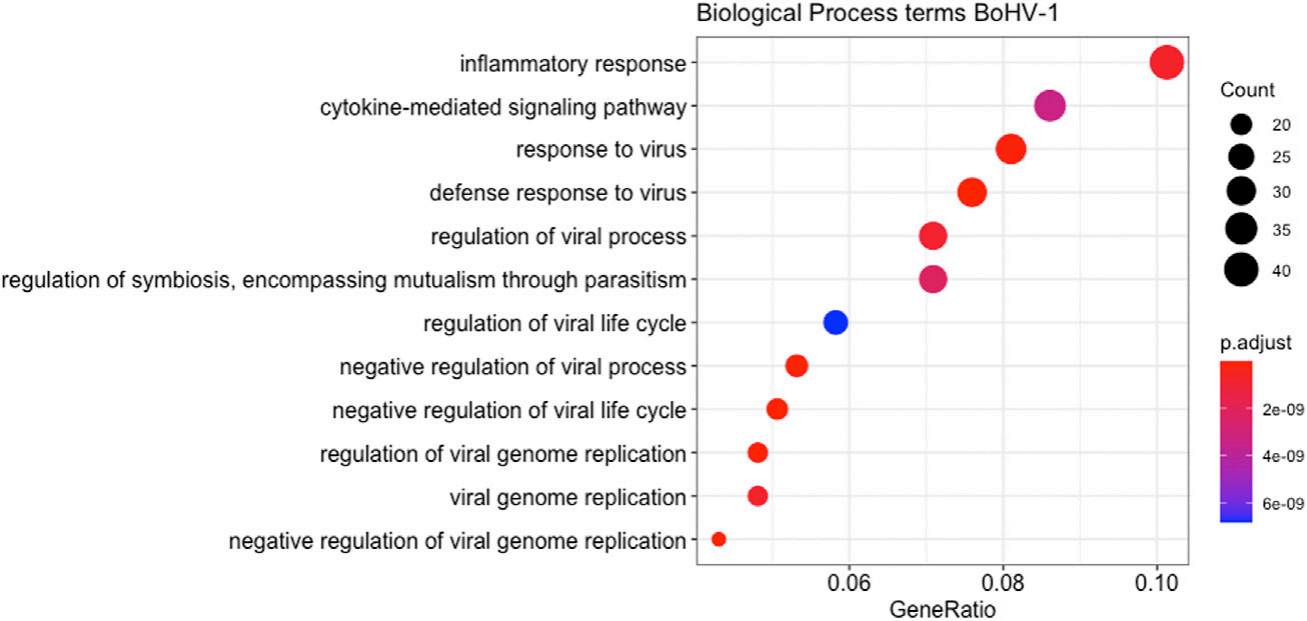
A dot plot displaying the statistically most significant 12 Biological Process GO terms enriched amongst the DEGs from the BoHV-1 challenge. GO term names are along the *Y*-axis while gene ratio is plotted on the *X*-axis. The colour relates to the level of significance of each term and the size of the bubble relates to the number of genes in each term.

Multi-dimensional scaling (MDS) showed a clear separation between the BoHV-1 challenged and control calves (Figure 2). There were 488 DEGs (*p* < 0.05, FDR <0.10, fold change ≥2) between the BoHV-1 challenged and control calves (Supplementary Table S1) (Figure 3), with the top five upregulated and downregulated genes outlined in Table 1. GO and enriched molecular pathway analyses of the DEGs using DAVID identified 13 KEGG pathways enriched for DEG between the BoHV-1 challenge and control calves (*p* < 0.05, FDR< 0.05), with the top five most significant pathways including Coronavirus disease–COVID-19, Influenza A, Cytokine-cytokine receptor interaction, *Staphylococcus aureus* infection and Hepatitis C. (Figure 4) (Supplementary Table S2). The DAVID analysis also identified 100 significant Biological Process, 19 Molecular Function and 5 Cellular Component (*p* < 0.05, FDR <0.05) GO terms (Figure 5) (Supplementary Table S3).

**TABLE 1:** A table showing the top 5 upregulated and top 5 downregulated genes identified in challenged calves in response to BoHV-1. Gene name; gives the name and corresponding gene symbol of each gene, Log 2fold change; contains the log fold change value or each gene with a positive and negative value for upregulated and downregulated genes respectively, *p*-value; presents the significance of each gene (*p* ≤ 0.05), Function; Gives a prospective role or function of each gene.

Gene name	Log 2fold change	*p*-value	Function
*ENSBTAG00000031825* ^a^	9.6	2.15 × 10^−8^	–
*PRSS2 serine protease 2*	5.23	7.27 × 10^−6^	Plays a role in inflammatory conditions e.g., pancreatitis
*CCL8*	5.11	9.37 × 10^−29^	Involved in monocyte attraction
*C-C motif chemokine 8*			
*IFI27*	5.06	6.25 × 10^−29^	Cytokine signalling and innate immune system
*Interferon alpha inducible protein 27*			
*ADM*	5.03	2.58 × 10^−24^	Role in antimicrobial activity
*Adrenomedullin*			
*DAB2*	−2.87	3.16 × 10^−19^	May act as a tumour suppressor
*DAB adaptor protein 2*			
*ENSBTAG00000013305* ^a^	−2.94	2.17 × 10^−10^	–
*ALAS2*	−3.60	7.86 × 10^−25^	Involved in haem synthesis
*5′ Aminolevulinate Synthase 2*			
*ADAMDEC1*	−3.91	1.13 × 10^22^	Dendritic cell function
*ADAM like decysin 1*			
*HBA*	−4.15	1.44 × 10^−13^	Oxygen transport from the lung
*Haemoglobin subunit alpha*			

^a^ Genes are not annotated.

**FIGURE 6:**
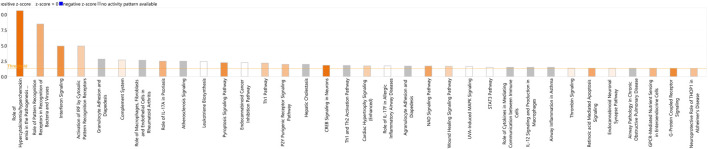
A bar-plot displaying the most significant canonical pathways identified amongst the DEGs in the BoHV-1 data (*p* < 0.05, FDR <0.05). Pathways are shown on the *X*-axis and the –Log Benjamini–Hochberg adjusted *p* values are displayed on the *Y*-axis. The threshold is set to 1.3, which equals a Benjamini - Hochberg adjusted *p*-value of 0.05. The orange line representing the ratio corresponds to the number of DE genes that map to the pathway over the total number of genes that map to the same pathway.

In addition, DEGs were analysed using Qiagen’s IPA platform where 34 canonical pathways were found to be enriched (*p* < 0.05, FDR <0.05) (Figure 6) (Supplementary Table S4). The most statistically significant of these pathways included Role of Hypercytokinemia/hyperchemokinemia in the Pathogenesis of Influenza, Role of Pattern Recognition Receptors in Recognition of Bacteria and Viruses, Interferon Signalling, Activation of IRF by Cytosolic Pattern Recognition Receptors, and Granulocyte Adhesion and Diapedesis.

Upstream regulator analysis (URA) in IPA aims to identify molecules upstream of DEGs that can potentially provide an explanation for the observed gene expression patterns within the data (Krämer et al., 2014). The top five most significant upstream regulators identified by URA were lipopolysaccharide, *NONO*, Interferon alpha, *IFNG* and *IFNL1* (Supplementary Table S5).

### Differential gene expression and pathway analyses of re-analysed BRSV RNA-Seq data

Following re-analysis, there were 306 DEGs (*p* < 0.05, FDR <0.10, fold change ≥2) (Supplementary Table S6) identified between the BRSV challenge and control calves. Similar to Johnston et al. (2021) an MDS plot revealed a clear separation between the control and challenged animals based on global gene expression (Supplementary Figure S1). From the DAVID analysis, there were six enriched KEGG pathways for the DEGs (*p* < 0.05, FDR <0.05), (Supplementary Table S7), and a total of 28 enriched GO Biological Process terms (*p* < 0.05, FDR <0.05) (Supplementary Table S8).

**FIGURE 7:**
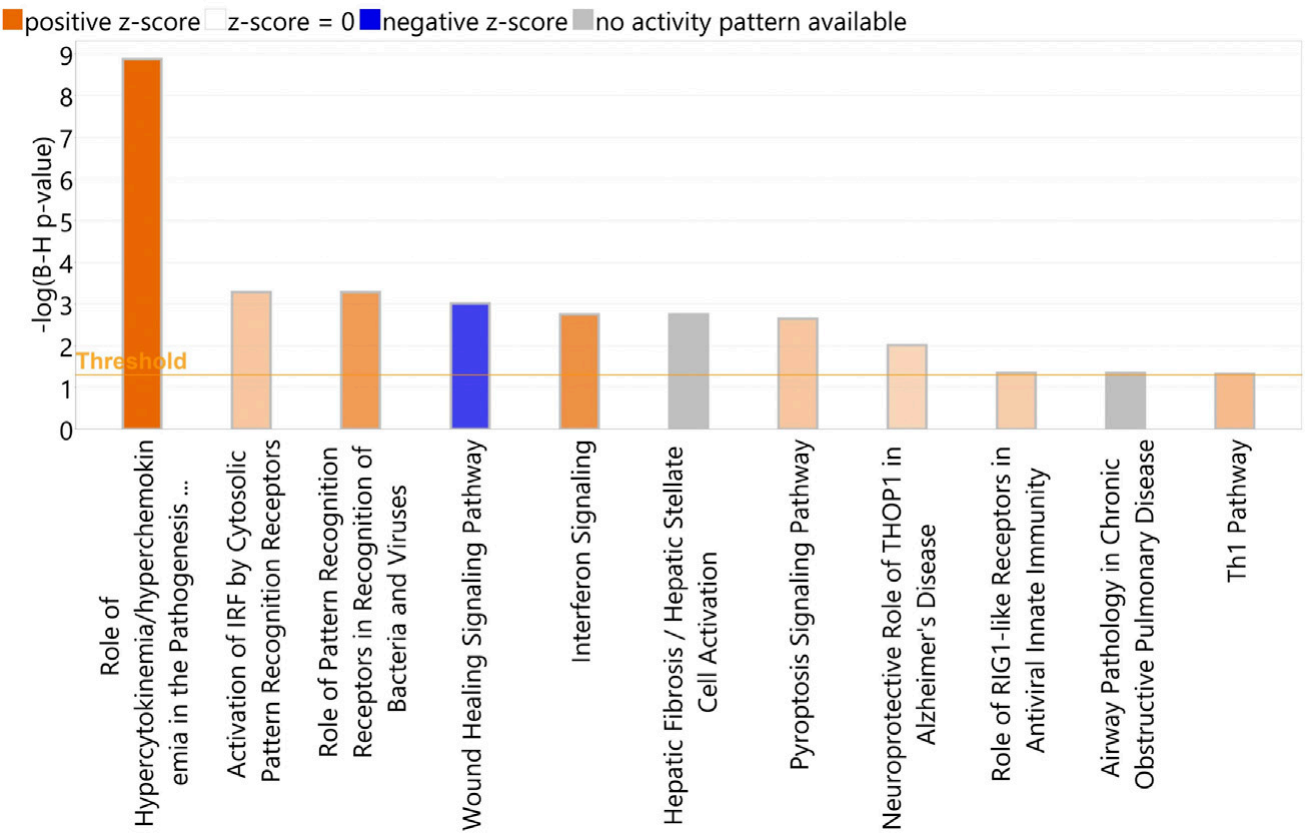
A bar-plot displaying the most significant canonical pathways identified amongst the DEGs in the re-aligned BRSV data (*p* < 0.05, FDR <0.05). The pathways are shown on the *X*-axis and the –Log Benjamini–Hochberg adjusted *p* values are displayed on the *Y*-axis. The threshold is set to 1.3, which corresponds to a –Log Benjamini—Hochberg adjusted *p*-value of 0.05.

IPA identified a total of 11 enriched canonical pathways (*p* < 0.05) with the most statistically significant pathways including Role of Hypercytokinemia/hyperchemokinemia in the Pathogenesis of Influenza, Activation of IRF by Cytosolic Pattern Recognition Receptors, Role of Pattern recognition Receptors in the Recognition of Bacteria and Viruses, Wound Healing Signalling Pathway, and Interferon Signalling (Figure 7) (Supplementary Table S9). The most statistically significant five upstream regulators identified in IPA were *NONO*, Interferon alpha, lipopolysaccharide, *IFNL1* and *IRF7* (Supplementary Table S10).

A comparison of the analysis using the UMD3.1 reference genome described by Johnston et al. (2021) and the re-analysis here using the ARS-UCD1.2 reference genome found that 222 genes were found in common in both analyses. However, there were 84 identified DEG unique to the ARS-UCD1.2 analysis and 59 DEG unique to the UMD3.1 analysis performed by Johnston et al. (2021) respectively (Supplementary Figure S2; Supplementary Table S11).

### Comparison of BoHV-1 and BRSV results

**FIGURE 8:**
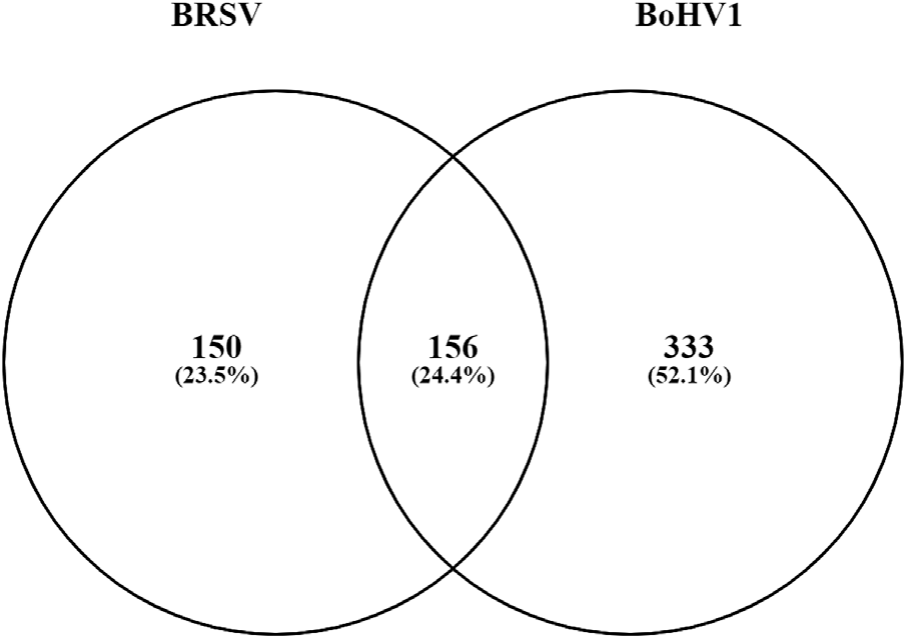
A Venn diagram outlining the commonality between the DE genes in the data from the current study and the data from Johnston et al. (2021). The circle labelled BoHV-1 represents the DEGs (333) unique to the BoHV-1 data and the circle labelled BRSV shows the number of DEGs unique to the BRSV data (150). The number of genes common to both studies (156) are in the overlap.

There were a total of 156 DEGs common to both the BoHV-1 and BRSV data (Figure 8; Supplementary Table S12). These genes were enriched by a factor of 13.53 compared to expectation under chance alone (*p* < 1.65e-145). Of these, 152 had the same direction of effect (Figure 7; Supplementary Table S12). Pathway analysis of the common DEGs and that had the same direction of effect (upregulated or downregulated) identified eight enriched KEGG pathways (*p* < 0.05, FDR <0.05) (Supplementary Table S13). These genes had 13 and 4 enriched Biological Process and Molecular function gene ontology terms, respectively (*p* < 0.05, FDR <0.05) (Supplementary Table S14).

Three hundred and thirty-three genes were unique to the BoHV-1 specific data. Analysis in DAVID identified 4 KEGG pathways, 11 Biological Process, 5 Cellular Component, and 2 Molecular Function ontology terms to be enriched (*p* ≤ 0.05, FDR ≤0.05) (Supplementary Table S15). Similarly, there were a total of 150 DEGs exclusive to the BRSV data and analysis showed 3 enriched Molecular Function ontology terms (*p* ≤ 0.05, FDR ≤0.05) (Supplementary Table S16). There were no enriched KEGG pathways, Biological Process or Cellular Component ontology terms enriched at the above thresholds for the BRSV specific data.

Following a comparative analysis in IPA, several differences in the activation and inhibition of canonical pathways were observed. The statistically most significant pathway possessing a difference in activation state across the two datasets was Pulmonary Fibrosis idiopathic signalling pathway, which was predicted to be inhibited (Z score < −2) in BRSV infection and activated (Z score >2) due to BoHV-1 infection (Supplementary Table S17). The upstream regulator analysis (URA) revealed similar regulators were predicted to be activated and deactivated across the two pathogen models (Supplementary Table S18). Additionally, identified diseases and functions were similar across the two datasets (Supplementary Table S19). (Supplementary Figure S3).

## Discussion

We examined changes in gene expression in circulating whole blood following a controlled experimental challenge with BoHV-1 in dairy calves. The DEGs identified in this study could provide information on potential therapeutic targets for BRD infection, and the comparison to Jonston et al. (2021) has highlighted the similarities and differences in the host response to each of the pathogens. With evidence of a specific host response for individual BRD pathogens (Tizioto et al., 2015; Behura et al., 2017), this comparison provides valuable information that could aid in disease prevention and treatment.

### Clinical signs and haematological variables

Challenged animals displayed clinical signs of BRD similar to observations reported by Gershwin et al. (2015). We found decreased WBCs in the BoHV-1 challenged animals, which is a common finding for viral infections (Roland et al., 2014). A shift in the population of WBC components (lymphocytopenia and neutrophilia) is also commonly observed during viral (Braun et al., 2018) and bacterial infections (Braun et al., 2021). Furthermore, BoHV-1 often infects lymphocytes, primarily CD4^+^ T cells, causing the induction of apoptosis (Jones, 2019). Neutrophilia, an increase in neutrophil number, is often witnessed during inflammation associated with respiratory tract infections (Roland et al., 2014) and, as found here, Lindholm-Perry et al. (2018) found that BRD-affected cattle exhibited greater neutrophil numbers than did asymptomatic controls. Neutrophils are key components of infection elimination and play a role in the pathogenesis of BRD through tissue damage and inflammation (McGill and Sacco, 2020). Neutrophils are relatively short-lived cells with a half-life of 8.9 h in the circulation which can be substantially increased during an inflammatory response (Paape et al., 2003). Consequently, it is possible that neutrophils need to have elements of the immune response actively transcribed even when inactive (i.e., circulating and not fighting infection). In the present study, it is likely that these genes then showed up in GO analyses. Clinical scores amongst the challenged animals were greater than those observed in the non-challenged calves. The clinical signs, rectal temperature and haematological changes observed in the challenged animals indicate the successful establishment of infection following the BoHV-1 challenge. Furthermore, the clear separation observed between the challenged and control animals in the MDS plot further supports the presence of infection and the perturbation of gene expression patterns due to infection.

### Expression patterns in response to BoHV-1 are characteristic of the immune response to viral infection

Significant changes in gene expression were identified between control animals and animals challenged with BoHV-1 and the activated pathways were associated with the immune response. These changes in gene expression observed here could be attributed to changes in transcriptional activity or an altered cellular composition of blood samples (e.g., neutrophil signature). Changes in expression due to either one or both of these parameters may be mediated directly by pathogen-derived molecules or the action of secondary factors released by the host (e.g., cytokines). We have observed major differences in the cellular composition of blood samples on d 4 only post-infection, with no change by d 6 when samples were collected for transcriptomic profiling. Therefore, it is unlikely the changes in DEGs were related to leukocyte counts, but instead to the inflammatory response. Moreover, it would be important to determine in future studies whether transcriptional analysis of blood leukocytes can provide information that would permit following the temporal progression of the disease from d-1 post-infection.

Ontology terms such as defence response to virus and negative regulation of viral genome regulation were amongst the most significant and encompass genes such as *IFIT1, OAS1X, IFIT5, OAS2, IL12B, ISG15, MX1, IFITM3, RSAD2, OASIY, OAS1Z, ISG20, APOBEC32,* and *IFITM3.* Furthermore, enriched KEGG pathways were also related to viral infection with Influenza A amongst the most significant. Genes such as *STAT2, MX2, OAS2, DDX58, MX1, IRF7, CXCL8, CXCL10, EIF2AK2, OAS1X, RSAD2, OAS1Y,* and *OAS1Z* were involved. This is similar to the findings of Sun et al. (2020), where the expression of several genes (*ISG15*, *MX1,* and *OAS2*) were recommended as biomarkers for the recognition of sick animals at feedlot entry. Many of these genes were also DE in whole blood Johnston et al. (2021) and bronchial lymph node (Johnston et al., 2019) in response to BRSV infection, and some of these genes (*MX2, RSAD2, ISG20, ISG15,* and *OAS2*) were identified as DE between beef cattle that contracted BRD and survived *versus* those that contracted the disease and died, with all of the above named genes being downregulated in animals that survived (Scott et al., 2021). Since *OAS1, OAS2, OAS1X, OAS1Y, OASZ, DDX58, STAT2, MX1,* and *MX2* are associated with the innate immune response to viral infection, it is not surprising that they were found to be upregulated in the challenged animals in this study.

The most upregulated gene identified in response to BoHV-1 was unannotated, (ENSBTAG00000031825). However, this gene was found to be highly upregulated in an *in vivo* study examining the transcriptomes of embryos from high or low field fertility Norwegian red bulls (Diaz-Lundhal et al., 2021). One suggested function of this gene was related to lipid metabolism, although the exact cellular mechanism is poorly understood (Mignani et al., 2020). This gene was also differentially expressed between Holstein and Jersey calves, on the day of weaning initiation, in a study investigating the effect of breed and gradual weaning on the whole blood mRNA transcriptome of artificially reared Holstein-Friesian and Jersey calves (Johnston et al., 2016). The finding of this gene in the present study may be useful towards deciphering what biological functions it has in bovine disease.

The finding of enrichment of DEGs within pathways associated with human viral diseases such as COVID-19 reflects the extent of research conducted since the pandemic onset and the influx of these data to gene ontology platforms such as DAVID. Clearly, the discovery of these pathways does not indicate that these animals were infected with COVID-19 or Hepatitis C but reflects the common involvement of genes in these viral pathways to the immune response to a broad spectrum of viruses. Many of these genes are associated with the inflammatory response. Genes within these pathways include *MX1, MX2, DDX58, CXCL10, OAS2, OAS1X, OAS1Y, OAS1Z, STAT2*, and *EIF2AK2*, and several of these (*MX1, MX2, OAS2, OAS1Y, ISG15, DDX58*, and *EIF2AK2*) have a potentially important role in BRD infections (Wang et al., 2014).

The activation and migration of leukocytes to sites of infection is often the first step in the host immune response (Hasankhani et al., 2021). Granulocyte Adhesion and Diapedesis was among the statistically most significant of the canonical pathways identified by IPA and genes involved include *CCL8, CD99, CLDN3, CXCL5, CXCL8, CXCL10, GNAI1, IL18, IL1R2, MMP14, PPBP, SDC3*, and *SELP*. This pathway is involved in leukocyte migration to sites of injury or infection. Additionally *CFB, CLEC5A, CLEC6A, DDX58, IFIH1, MX1, PML, RSAD2, S100A8*, and *TLR5* were identified as key immune response genes in lung, lymph node and pharyngeal tonsil tissue in beef cattle challenged with BoHV-1 (Behura et al., 2017). The DEGs involved in the activation of these pathways in response to BoHV-1 could be useful BoHV-1 diagnostic target genes or could serve as potential therapeutic targets for BRD treatment.

### Inflammatory response is a key theme amongst the observed gene expression changes in response to BoHV-1

BoHV-1 has been shown to induce inflammation in calves during acute infection (Wang et al., 2014). Here, the inflammatory response was evident across enriched GO terms such as Inflammatory response, tumour necrosis factor and interleukin 6 production. Interleukin 6 is a pro-inflammatory cytokine produced in response to infection and tissue damage (Johnson et al., 2013). Expression patterns observed in the current study show the upregulation of multiple interferon stimulated genes such as *ISG15*, *OAS2*, *OAS1X*, *OAS1Y*, *OAS1Z*, *MX1,* and *MX2*. Although we did not find an altered expression of *IL-6*, the expression patterns suggest that this gene played a role in the immune response to BoHV-1. The detection of pathogens relies on the action of specific pattern recognition receptors (PRRs) such as NOD-like receptors. Here, the pathway for NOD-like receptor signalling was identified for the DEGs in response to BoHV-1 with genes involved including *STAT2, GBP4, GBP2, GBP5, NOD1, OAS2, CXCL8, CAPS4, IL18, IRF7, OAS1X, OAS1Y,* and *OAS1Z*. NOD-like receptors are involved in the regulation of inflammatory responses through the induction of immune associated genes such as cytokines and chemokines (Luo et al., 2018), a pattern found in the current data. In addition, the Cytokine-Cytokine receptor interaction KEGG pathway was identified and includes genes such as *CXCL8, MPL, CSF1, IL12B, IL5RA, IL1R2, IL10, IL18, TNFSF9, TGFB2, CXCL10,* and *CCL8*. Cytokines are involved in host innate and adaptive immune responses and an increase in the expression of pro-inflammatory cytokines is often observed during viral infections (Saxena and Yeretssian, 2014). Evidence so far suggests that the inflammatory response is a key driver of subsequent immune processes in response to BoHV-1 infection.

### The role of serine protease genes in the immune response to BoHV-1

The KEGG pathways for Complement and coagulation cascades and platelet activation were enriched for the DEGs in response to BoHV-1 infection. Interestingly, these pathways were not enriched for the DEGs found in the BRSV data. The complement and coagulation pathways consist of three protein networks (complement system, coagulation cascade, and fibrinolytic system, respectively) and are involved in the innate immune response (Mogensen and Paludan., 2001). The complement system acts as an immune surveillance system (Oikonomopoulou et al., 2012) and coagulation is involved in blood clotting, primarily at sites of vascular injury (Stoermer and Morrison., 2011). A key feature of these systems is that they all involve the activity of serine proteases, the largest class of mammalian proteases, which play a role in coagulation, fibrinolysis and complement activation (Owen, 2006). Pathway genes identified in this study include *C4A, C2, PROCR, F2RL2, C1R, CFI, CFB, C3AR1, CLU*, and *VWF*. Tizioto et al. (Tizioto et al., 2015) also found this pathway to be enriched for DEGs in response to an IBR challenge in beef cattle with many of the same genes being involved. Interestingly, we found serine protease 2 (*PRSS2*) to be the one of the most highly upregulated genes in response to BoHV-1. Although this gene is not directly involved in these pathways, *PRSS2* is associated with inflammatory conditions in humans such as pancreatitis (Witt et al., 2006) and inflammatory bowel disease (Wang et al., 2021). Furthermore, high levels of expression of serine proteases have been identified in cases of lung disease in both human and animal models (Owen, 2006). Although the complement and coagulation pathways are vital for a robust immune response, their excessive activation can be harmful and lead to excessive clotting (Antoniak and Mackman, 2014) as well as heightened inflammation and clinical symptoms of disease (Ricklin and Lambris, 2013). Interestingly, serine proteases are crucial for the replication of herpesviruses and protease activity is key in the final stages of the virus lifecycle, specifically for capsid assembly and DNA packing (Skoreński et al., 2016). The role of serine proteases in response to BoHV-1 could serve as a useful future research direction to aid in the development of therapeutics for BoHV-1 infection.

### Re-analysis of the BRSV data using the ARS-UCD1.2 reference genome

Improvements in long read sequencing technologies and new scaffolding methodologies allowed for the assembly of the ARS-UCD1.2 reference genome in 2020 (Rosen et al., 2020). A comparison to the previous UMD3.1 reference genome found that that the ARS-UCD1.2 assembly was significantly improved based on an analysis of the structurally complex major histocompatibility complex (MHC) region (Zorc et al., 2019). The re-alignment of the BRSV RNA-Seq data from Johnston et al. (2021) to the updated ARS-UCD1.2 reference genome resulted in the identification of an additional 8.9% (25) DEGs compared to the previous analysis described in Johnston et al. (2021), which is primarily due to the improved assembly and annotation of the ARS-UCD1.2 reference genome. The current analysis uses the same package (EdgeR) and statistical thresholds as those used previously by Johnston et al. (2021). Furthermore, MHC genes such as *BOLA-DQB* were found to be more highly DE in the re-analysis, which could be a result of the improved assembly of this gene (Zorc et al., 2019). Examination of the genes common and unique to each analysis (i.e., using the UMD3.1 vs. the ARS-UCD1.2 assembly) showed that 84 and 59 DEGs were unique to the ARS-UCD1.2 and UMD3.1 analyses respectively. This large difference could be due to the small sample size used in this study. A comparison of analyses with the different assemblies should be repeated using a larger dataset with more replicates in order to validate these findings. Non-etheless, it is interesting to see the differences between the annotations, and our findings suggest that the re-analysis of existing RNA-Seq data against the ARS-UCD1.2 reference assembly could provide further insights into gene expression profiles.

### Similarities and differences in the host response to BoHV-1 and BRSV

The KEGG pathway for Influenza A was among the most statistically significant pathways identified in response to single pathogen infection with BoHV-1 and BRSV. This pathway was also one of the most significant identified in the bronchial lymph nodes of dairy calves challenged with BRSV (Johnston et al., 2019). Interestingly, although some of the same pathways were identified in both the BoHV-1 and BRSV data, a comparison of the pathway-associated genes revealed the involvement of different genes in pathway activation. Focusing on the Influenza A pathway, which was highly enriched in both datasets, there were 20 (*IFIH1, PML, STAT2, MX2, OAS2, CXCL8, IL12B, PRSS2, DDX58, MX1, ADAR, IL18, IRF7, CXCL10, EIFAK2, OAS1X, RSAD2, ENSBTAG00000037605, OAS1Y*, and *OAS1Z*) and 16 (*IFIH1, BOLA-DQA5, PML, MX2, OAS2, CXCL8, IL12B, DDX58, MX1, BOLA-DQB, IRF7, CXCL10, EIF2AK2, RSAD2, OAS1Y*, and *OAS1Z*) genes involved in the Influenza A pathway in the BoHV-1 and BRSV datasets, respectively. Some key differences in genes include the MHC genes, *BOLA-DQ5*, and *BOLA-DQB* involved in this pathway that were DE in response to BRSV but not to BoHV-1. It is interesting that although similar pathways are at work in each challenge model, the genes governing the activation of these pathways can vary based on the specific pathogen with similar phenomena observed in similar studies using beef cattle (Tizioto et al., 2015; Behura et al., 2017). These differences in genes despite the same pathway being activated, further strengthens the idea of a pathogen-specific immune response.

Many genes were found in common for both challenge models such as *IFI27*, *ADM*, *CCL8*, and *SIGLEC1*, which were amongst the 10 most significantly upregulated genes in response to BoHV-1 and BRSV. *CCL8*, and *ADM* are involved in the inflammatory response (Wong et al., 2005; Lisboa et al., 2015). *IFI27* is an interferon induced gene and is suggested to have an antiviral function against Hepatitis B virus (Ullah et al., 2021). *SIGLEC1* is also involved in the immune response to viral infection and is upregulated following the detection of type 1 interferons (Perez-Zsolt et al., 2019). Also common to both models was the downregulation of *ADAMDEC1*, which is involved in the maturation of dendritic cells that play a role in the adaptive immune system and may be associated with immunity (O'Shea et al., 2016). Furthermore, Epstein–Barr virus (EBV) (a herpesvirus) has been shown to induce the suppression *ADAMDEC1* which contributes to the formation of EBV-associated cancers (Gillman et al., 2018).

Analysis of the DEGs common to both the BoHV-1 data and the BRSV data from Johnston et al. (2021) identified the KEGG RIG-I-like receptor signalling pathway as the most significant. Genes involved in this pathway include *IFIH1, CXCL10, CXCL8, DDX58, DHX58, IRF7, IL12B*, and *ISG15*, which, as mentioned above, play a role in the immune response. RIG receptor signalling is involved in viral recognition and cell recruitment for viral elimination, through the production of type 1 interferons and inflammatory cytokines (Kawai and Akira, 2008). Most significant GO terms amongst these common DEGs include immune response and defence response. Genes involved in these pathways and GO terms common to both pathogen responses are identified here as potential targets for BRD treatment strategies.

Despite these similarities, there were several differences between the pathogens. The most strongly downregulated gene in response to BoHV-1 was *HBA* or *haemoglobin subunit alpha*, which is involved in the transport of oxygen from the lungs to various peripheral tissues (The UniProt, 2021). This gene, amongst other haemoglobin genes were also found to be downregulated in beef cattle diagnosed with BRD (Jiminez et al., 2021). This is in contrast to findings for the BRSV challenge model where the collagen genes *COL1A1* and *COL1A2* were downregulated by −18 and −24 fold respectively, which is similar to the findings of Johnston et al. (2021).

Lipopolysaccharide (LPS) was identified as the most significant upstream regulator in the current data and was predicted to affect the expression patterns of 142 genes in these data. This molecule was also identified as one of the most significant upstream regulators in bronchial lymph nodes isolated from Angus-Hereford calves experimentally challenged with BoHV-1 and BRSV (Tizioto et al., 2015) and those isolated from Holstein-Friesian calves experimentally challenged with BRSV (Johnston et al., 2019). LPS induces a cascade of both anti-viral and anti-bacterial genes and has previously been suggested as a treatment target for the prevention of BRD infections (Tizioto et al., 2015). In contrast, LPS has been shown to accelerate the rate of herpesvirus 1 replication in human epithelial cells. This may indicate that LPS could play a similar role in BoHV-1 infections in cattle and could be a fruitful area for further research. Although LPS was amongst the five most significant upstream regulators in the BRSV data, *NONO* (Non-POU Domain Containing Octamer Binding) was the most significant, targeting 36 genes within the BRSV data, including several immune associated genes (*CCL8*, *CXCL10*, *DDX58*, *IFI27*, *ISG15*, *OAS1*, and *OAS2*). *NONO* has previously been identified as an important sensor in HIV infections and is responsible for innate immune activation in key immune cells (Lahaye et al., 2018).

Taking the DEGs identified in response to each of the respective pathogens, there were a 333 and 150 genes unique to the BoHV-1 and BRSV specific data respectively. Pathway and gene ontology analysis of these unique genes uncovered similar findings to those seen in the above DEG analysis. For the genes unique to BoHV-1, the most significant pathway was “Platelet Activation” with genes including *GP5, P2RY1, MYLK, ADCY8, GP9, GNAI1, ADCY3, GP1BA, P2RX1*, and *VWF* involved. Platelets and their products are often regarded as a “double-edged sword” during viral infections as they can be involved in infection suppression or, in certain cases, can aid the viral infection (Assinger, 2014). Although analysis of the BRSV specific genes failed to identify significant KEGG pathways at the thresholds used, GO terms identified may aid in further understanding the specific host response to BRSV. The genes unique to each pathogen as well as their associated pathways and ontologies further highlight the biological processes that are involved in each of the respective models.

We acknowledge that the diagnosis of BoHV-1 associated BRD typically relies on, either alone or in combination with clinical signs, serology, culture, PCR, gross pathology and histopathology.

Since we have not ruled out a secondary infection of the lung tissue in the present study, with 100% certainty, we therefore cannot attribute the DEG changes solely to BoHV-1. Work is ongoing to characterise bacterial populations in the lung from animals in the current study to uncover their role during the course of BoHV-1 induced infection. The “traditional” model of BRD pathogenesis proposed a primary role for viral agents (and some bacterial pathogens) disabling host defences thereby facilitating secondary bacterial proliferation and associated lung pathology. Recently this “traditional” model has been challenged through the recognition that certain pathogens can act in either a primary or secondary role (Gagea et al., 2006; Nikunen et al., 2007) and that other stressors such as transportation, housing, weaning and other environmental factors can play a central role in mediating the impairment of respiratory tract defences. The ongoing microbiome analyses will thus provide an insight into the dysbiosis of the bacterial population during the course of BoHV-1 viral infection and identify possible secondary BRD pathogens.

## Conclusion

Besides identifying genes that are DE in the whole blood of dairy calves in response to a key BRD-causing virus, BoHV-1, this study has identified genes that are uniquely DE in response to specific BRD causing pathogens, through a comparison of two separate challenge studies conducted using dairy calves. This study has shown, as have other studies, that although similar pathways are at play during the response to individual infections, there are several different genes for each of the pathogens governing the activation of these pathways. Moreover, detecting these gene expression changes in circulating whole blood highlights the potential for the development of a BRD diagnostic, for which no “gold standard” test exists. In contrast to other internal tissues, whole blood can be routinely collected from live animals, thus serving as a suitable “ante-mortem” tissue for use in diagnostic applications. Furthermore, whole blood collection is easier and less invasive than sampling internal tissues (Stoot et al., 2014). This, as also suggested by Johnston et al. (2021), has potential for the development of effective diagnostic tools. In future work, we will analyse the microbiota of the lung to determine if secondary bacterial agents play a role in BoHV-1. The DEGs and associated molecules identified in this study, could serve as potential therapeutic targets for the treatment of BRD. Additionally, highly DE genes, both unique to the respective viral pathogens and those common to both, could be examined in larger scale studies for the identification of variants potentially associated with differential susceptibility to BRD infection, which would assist in selective breeding for reduced disease susceptibility. Additionally the examination of responses during a combined BRSV and BoHV-1 challenge (co-infection) could be an interesting direction for future work.

## Data Availability

The datasets presented in this study can be found in online repositories. The names of the repository/repositories and accession number(s) can be found below: https://www.ncbi.nlm.nih.gov/geo/, GSE199108.
