# Supplementary material for: Whole blood transcriptome analysis in dairy calves experimentally challenged with bovine herpesvirus 1 (BoHV-1) and comparison to a bovine respiratory syncytial virus (BRSV) challenge
Source: Front Genet. 2023 Feb 17;14:1092877. doi: 10.3389/fgene.2023.1092877 (PMC9981960; doi:10.3389/fgene.2023.1092877)
Supplement: Supplementary file 1 [file DataSheet1.zip › Supplementary materials/Supplementary_figures.pdf]

Supplemental Information for:

Whole blood transcriptomic analysis in dairy calves experimentally challenged with BoHV-1 and comparison to a Bovine Respiratory Syncytial Virus (BRSV) challenge

Stephanie O'Donoghue[1,2], Bernadette Earley[1], Dayle Johnston[1], Matthew S. McCabe[1], JaeWoo Kim[4], Jeremy F. Taylor[4], Ken Lemon[3], S. Louise Cosby[3], Derek W. Morris[2], Sinead M. Waters[1*]

[1]Animal and Bioscience Research Department, Animal and Grassland Research and Innovation Centre, Teagasc, Grange, Co., Meath, Ireland

[2]Discipline of Biochemistry, National University of Ireland, Galway

[3]Veterinary Sciences Division, Agri-Food and Biosciences Institute, Stormont, Belfast, Northern Ireland

[4]Division of Animal Sciences, University of Missouri, Columbia, MO, USA

[*]Sinead.Waters@teagasc.ie

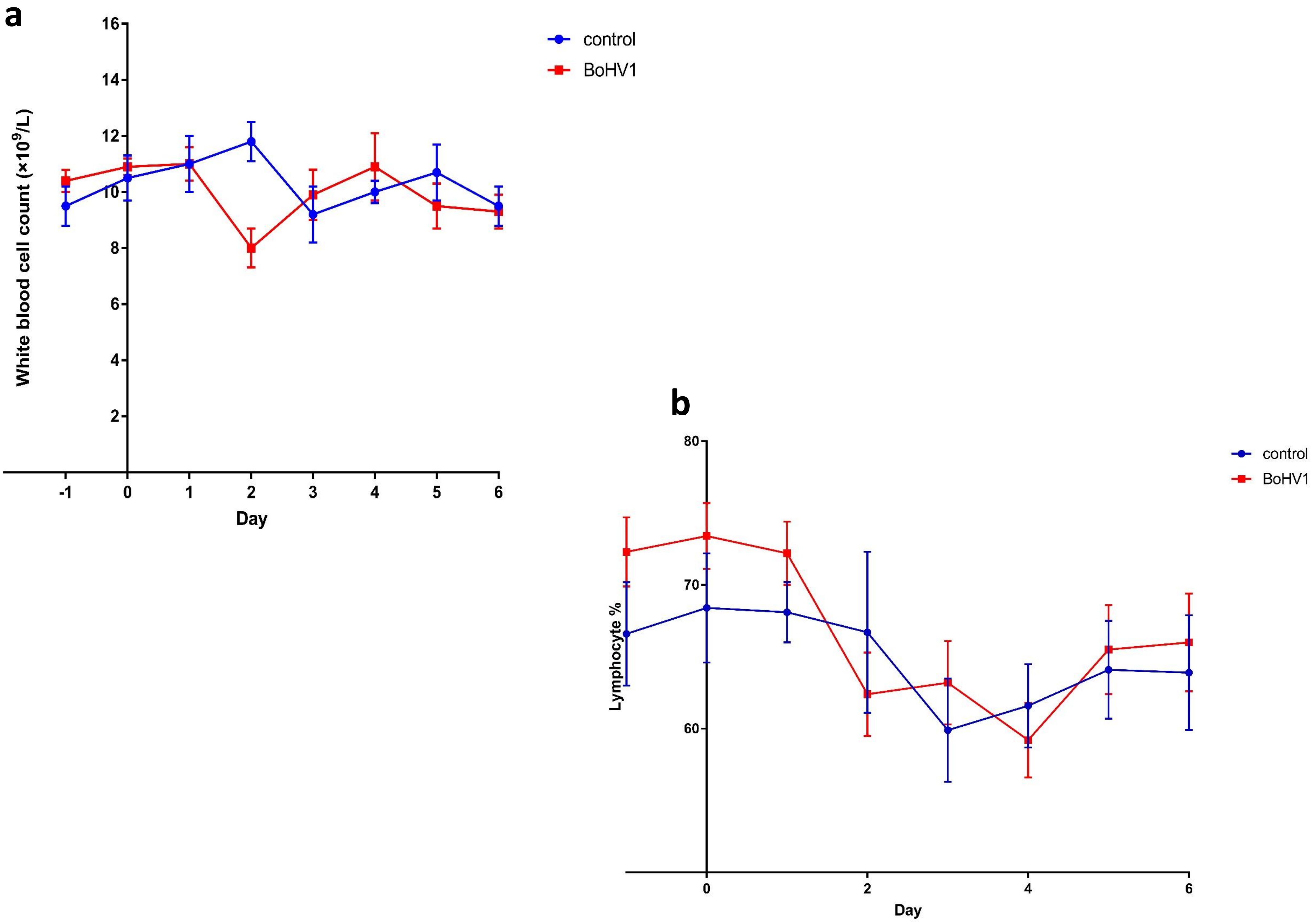

a
White blood cell count (×10⁹/L)
16
14
12
10
8
6
4
2
-1
0
1
2
3
4
5
6
Day
control
BoHV1
b
Lymphocyte %
80
70
60
0
2
4
6
Day
control
BoHV1

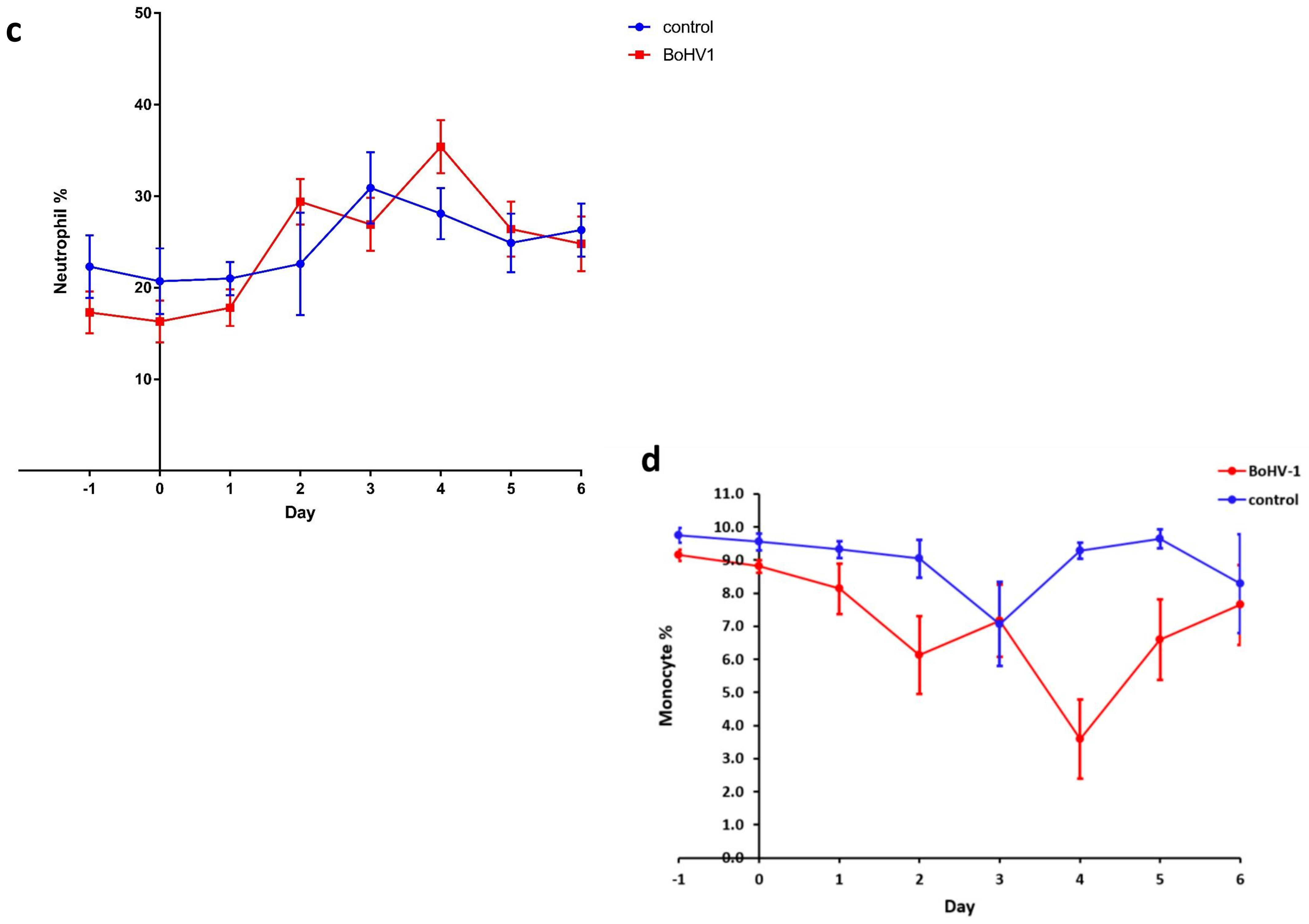
c
control
BoHV1
Neutrophil %
50
40
30
20
10
-1
0
1
2
3
4
5
6
Day
d
BoHV-1
control
Monocyte %
11.0
10.0
9.0
8.0
7.0
6.0
5.0
4.0
3.0
2.0
1.0
0.0
-1
0
1
2
3
4
5
6
Day

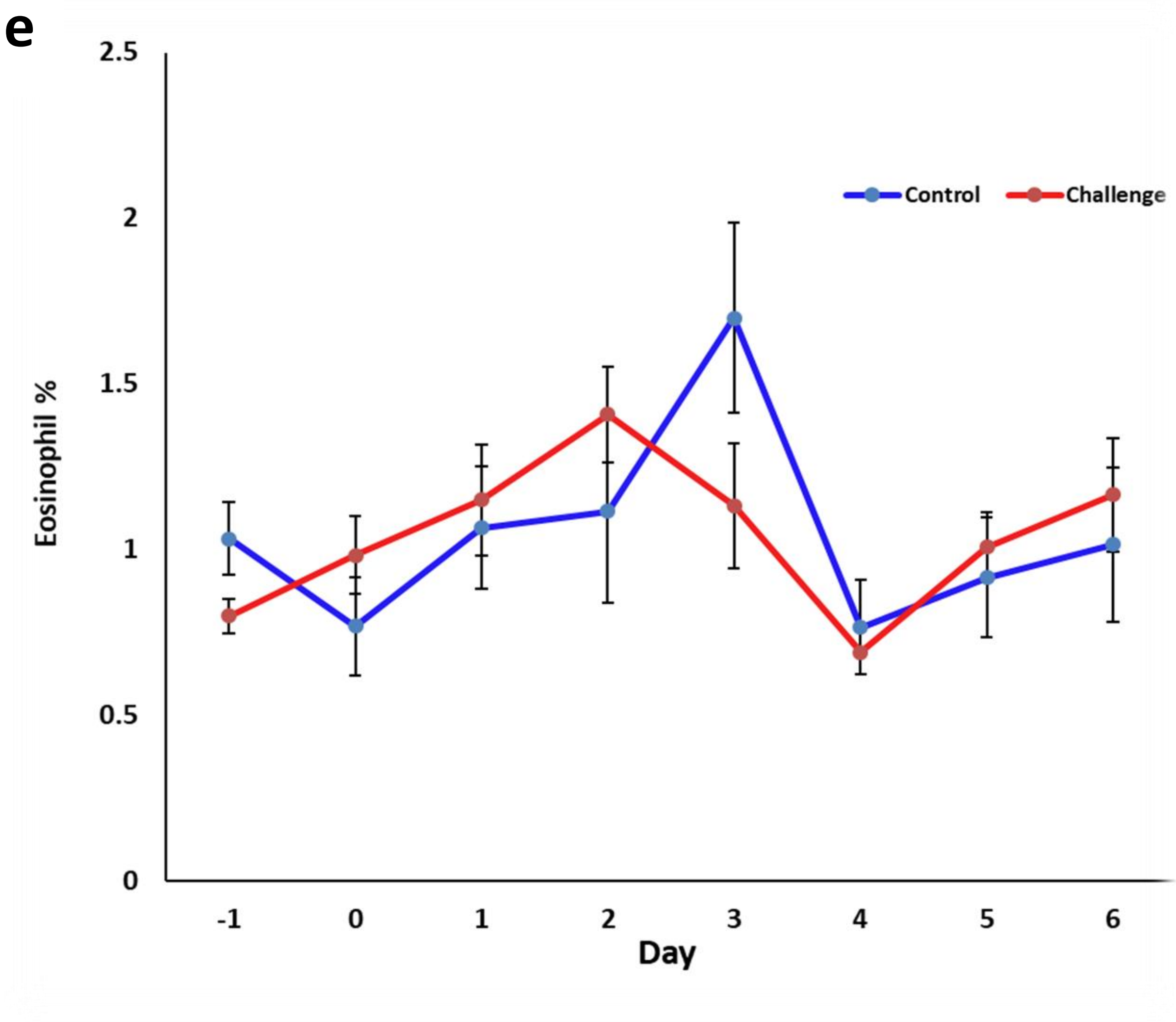

Supplementary Figure 1: Haematology counts for **a)** white blood cells, **b)** lymphcyte %, **c)** neutrophil %, **d)** monocyte % and **e)** eosinophil %.

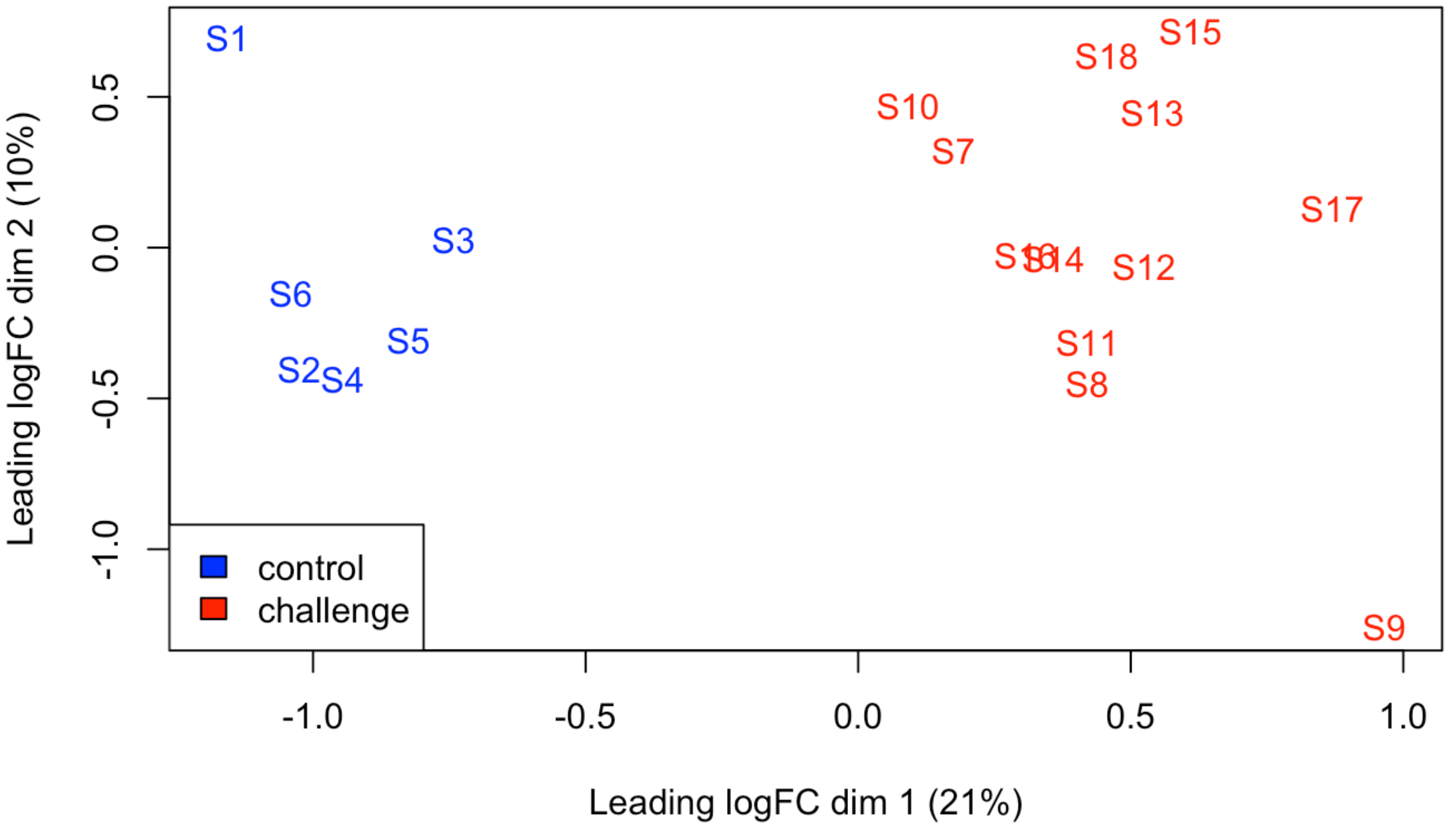

Supplementary Figure 2: An MDS plot generated using EdgeR in R displaying the similarity between the samples from Johnston et al. (2021) (BRSV challenge study). Samples from the Challenge calves are coloured red and Control samples are coloured blue. The numbers (1-18) refer to the calf ID. Sample (S); S1-S6 (Control) S7-S18 (Challenge).

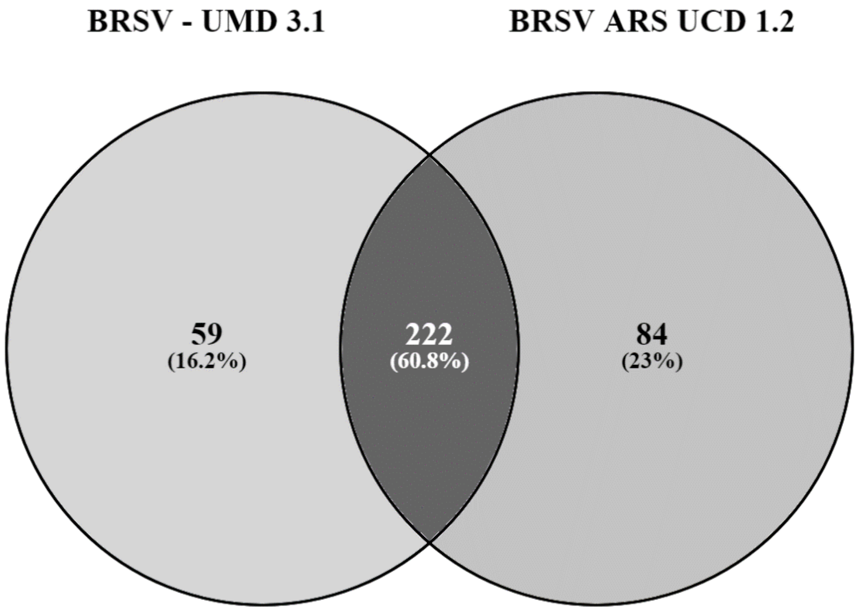

Supplementary Figure 3: A Venn diagram displaying the overlap of identified genes in the BRSV data between the UMD 3.1 and ARS UCD 1.2 bovine reference genome assemblies. There were 59 genes unique to the analysis using the UMD 3.1 assembly and 84 genes unique to the analysis using the ARD UCD 1.2 assembly. The darker area shows the number of genes common to both analyses, which was a total of 222 genes.

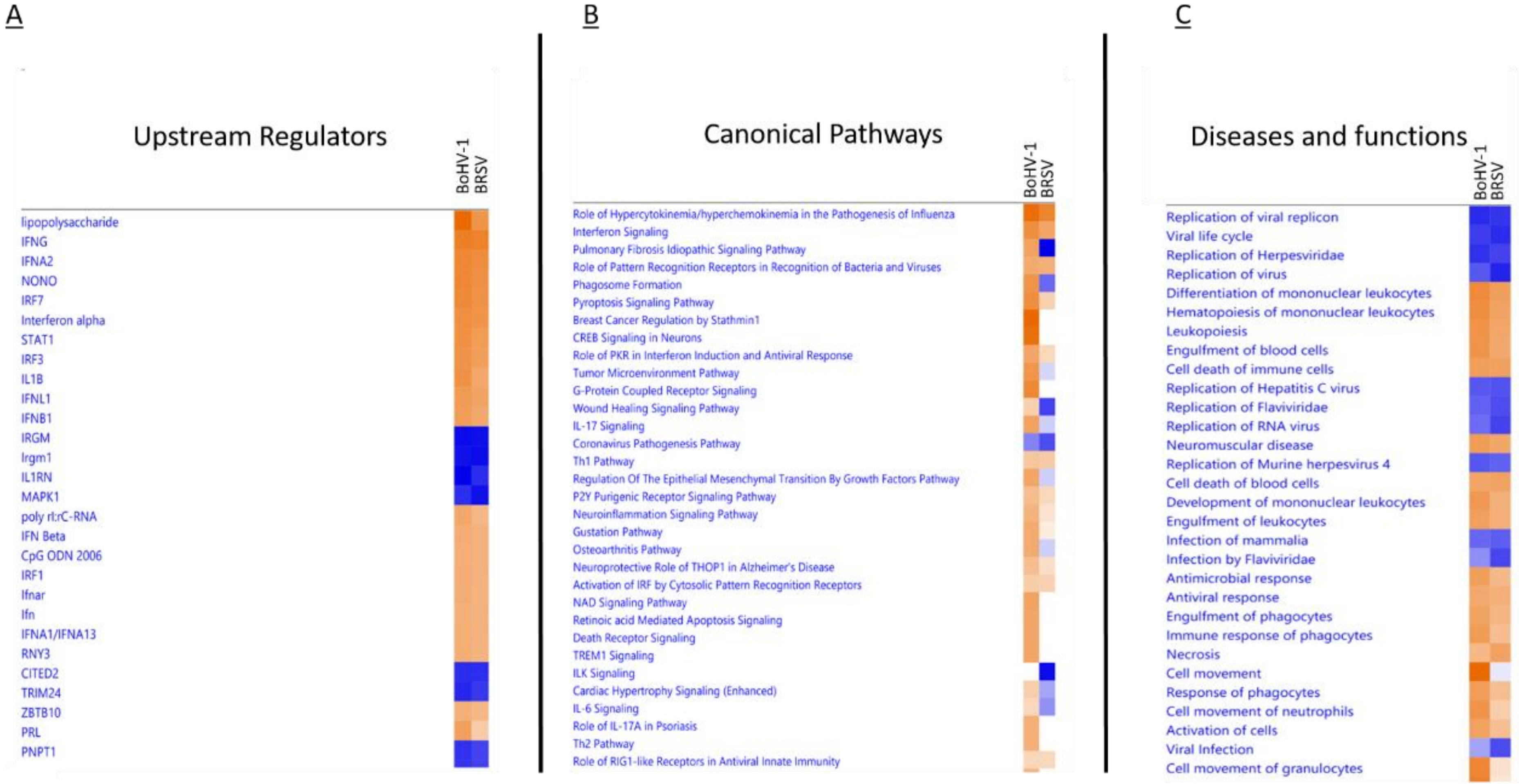

Supplementary Figure 4: Heat maps showing the results of the IPA comparison analysis between the BRSV and BoHV-1 datasets. The results of the canonical pathway, diseases & functions and upstream comparison analyses are shown in A, B and C respectively. The pathway names are written on the left, with the Z score result shown in the corresponding box on the right. The colour of the squares refers to the Z score (blue being a negative Z score and orange being positive).
